# Food from Equids—Commercial Fermented Mare’s Milk (Koumiss) Products: Protective Effects against Alcohol Intoxication

**DOI:** 10.3390/foods13152344

**Published:** 2024-07-25

**Authors:** Ming Du, Yuanyi Liu, Jialong Cao, Xinyu Li, Na Wang, Qianqian He, Lei Zhang, Bilig Zhao, Manglai Dugarjaviin

**Affiliations:** 1 Key Laboratory of Equus Germplasm Innovation, Ministry of Agriculture and Rural Affairs, Hohhot 010018, China; duming@imau.edu.cn (M.D.); 13470105913@163.com (Y.L.); 17647314067@163.com (J.C.); chinalxy94@163.com (X.L.); 15804814519@163.com (N.W.); qianqianhe202309@163.com (Q.H.); 13034784525@163.com (L.Z.); bilig9@163.com (B.Z.); 2 Inner Mongolia Key Laboratory of Equine Science Research and Technology Innovation, Inner Mongolia Agricultural University, Hohhot 010018, China; 3 Equus Research Center, Inner Mongolia Agricultural University, Hohhot 010018, China

**Keywords:** alcohol-induced harm, koumiss, mitochondrial and ribosomal functions, mouse model, protective effects

## Abstract

Fermented mare’s milk (koumiss), a traditional Central Asian dairy product derived from fermented mare’s milk, is renowned for its unique sour taste and texture. It has long been consumed by nomadic tribes for its nutritional and medicinal benefits. This study aimed to comprehensively analyze the protective effects of koumiss against alcohol-induced harm across behavioral, hematological, gastrointestinal, hepatic, and reproductive dimensions using a mouse model. Optimal intoxicating doses of alcohol and koumiss doses were determined, and their effects were explored through sleep tests and blood indicator measurements. Pretreatment with koumiss delayed inebriation, accelerated sobering, and reduced mortality in mice, mitigating alcohol’s impact on blood ethanol levels and various physiological parameters. Histopathological and molecular analyses further confirmed koumiss’s protective role against alcohol-induced damage in the liver, stomach, small intestine, and reproductive system. Transcriptomic studies on reproductive damage indicated that koumiss exerts its benefits by influencing mitochondrial and ribosomal functions and also shows promise in mitigating alcohol’s effects on the reproductive system. In summary, koumiss emerges as a potential natural agent for protection against alcohol-induced harm, opening avenues for future research in this field.

## 1. Introduction

Ethanol, as a structurally simple organic compound, can easily penetrate cell membranes and rapidly enter human cells. After consumption, ethanol enters the body, with approximately 2% being excreted through respiration, urine, and sweat. A portion is absorbed and metabolized in the gastrointestinal tract, while the majority (about 90%) is broken down in the liver via its metabolic system [1,2,3]. Ethanol metabolism in the liver primarily occurs through three pathways: the alcohol dehydrogenase system, the microsomal ethanol oxidizing system, and the peroxidase system [4,5]. These metabolic pathways effectively reduce the potential harm of ethanol to the human body, ultimately oxidizing acetate to CO_2_ and H_2_O. Despite the relatively well-understood metabolic mechanism of ethanol, alcohol abuse remains a significant global public health issue with severe health consequences that cannot be ignored. The World Health Organization has classified alcohol as a Group 1 carcinogen, indicating that long-term alcohol consumption can not only alter certain blood parameters but also cause substantial damage to organs such as the liver, stomach, and small intestine, with no effective treatment currently available [6,7,8]. This highlights the urgent need for exploring potential protective agents against alcohol-induced harm [9,10].

In this context, fermented mare’s milk (koumiss, airag, or chige), a dairy product with a unique flavor, has garnered significant attention owing to its rich nutritional value and medicinal properties. It appears milky white or light blue and is highly favored by nomadic tribes in Central Asia, Mongolia, Russia, and regions inhabited by Mongolian people [11]. Koumiss is distinguished by two notable characteristics: firstly, it is made from fresh mare’s milk, retaining original nutrients and supplemented with beneficial microorganisms, amino acids, trace elements, etc., making it easier for the body to absorb; secondly, it possesses special medicinal values such as improving spleen function, promoting digestion, enhancing physical strength, regulating qi and blood, and reducing blood lipid levels, blood acidity, and total cholesterol, as well as improving overall physical fitness [12,13,14,15]. These properties suggest that koumiss may have potential health benefits, particularly in mitigating the adverse effects of alcohol consumption. Given its potential, this study aims to investigate the protective effects of koumiss against alcohol-induced damage (behavioral, hematological, gastrointestinal, hepatic, and reproductive) by establishing a mouse model of acute alcohol intoxication. Furthermore, this study intends to explore the potential mechanisms underlying its impact on the reproductive system of intoxicated mice through transcriptome sequencing, with the ultimate goal of providing a theoretical basis for the further development and utilization of koumiss as a potential intervention for alcohol-related health issues.

## 2. Materials and Methods

### 2.1. Experimental Samples

SPF Kunming male mice (Spefo Biotechnology Co., Ltd., Beijing, China) that passed quarantine inspection, weighing 30–40 g, were kept in an environment with 12 h light and 12 h dark cycles (6:00–18:00 light). The ambient temperature was maintained at 20 ± 2°C, and humidity was kept at 50–60%. Except for fasting and the free drinking water 12 h before the experiment, they were free to eat and drink every day. All sampling procedures were approved by the Laboratory Animal Welfare and Ethics Committee of Inner Mongolia Agricultural University and met regulatory standards. The baijiu used in the mouse model of alcohol intoxication is a product produced by Beijing Red Star Co., Ltd., (Beijing, China) with an alcohol content of 43%. Its main ingredients include water, sorghum, barley, and peas. The fermented mare’s milk (koumiss) was purchased from Inner Mongolia Masu Dairy Co., Ltd., located within the compound of the Mongolian Medicine Comprehensive Hospital in Xilinguole Prefecture, Inner Mongolia, China. The product standard code is DBS15/013-2019, and the production license number is SC10515250201118 [16]. The detailed nutritional composition table is shown in Table S1.

### 2.2. Establishment of Mouse Alcohol Intoxication Model

The mice were randomly grouped and administered with different doses of 56° Red Star Erguotou (Red Star Co., Ltd., Beijing, China) by gavage. The disappearance of the righting reflex of the mice (righting reflex: if the mouse is gently rolled over and maintains a prone position for more than 30 s after drinking for a period of time, it can be judged that the righting reflex has disappeared) was used as the criterion for intoxication, and the phenomenon of the rapid respiratory rate of individual mice was used as an auxiliary criterion for intoxication. We observed and recorded the intoxication status of the mice after 30 min and the death status after 24 h, and then we screened for the optimal dose of Baijiu for gavage [17,18].

### 2.3. Determination of the Optimal Dose of Fermented Mare’s Milk for Gavage in Mice

Mice were randomly divided into five groups: control group (B), liquor group (L), low-volume koumiss group (LL), medium-volume koumiss group (ML), and high-volume koumiss group (HL). LL, ML, and HL groups were gavaged with koumiss (Inner Mongolia Masu Dairy Co., Ltd., Hohhot, China) according to the dosage of 0.05, 0.10, and 0.15 mL/10 g, respectively, and the interval between the two substances’ gavage was 30 min. The optimal dose of sour horse milk was determined by consecutive gavage for 7 days. The optimal amount of koumiss was screened [19,20].

### 2.4. Mouse Drunk Sleep Test

Fifty healthy male mice without abnormalities were randomly selected and fasted for 12 h before the experiment while allowing water intake. They were divided into five groups according to the randomized block design: control group (B), liquor group (L), koumiss then liquor group (KL), liquor then koumiss group (LK), and liquor then saline group (LS), with 10 mice in each group. The liquor was administered at a dose of 0.16 mL/10 g, while normal saline and koumiss were administered at a dose of 0.10 mL/10 g. The interval between the administration of the two substances was 30 min. The disappearance of the righting reflex in mice was used as the primary indicator of intoxication, and the phenomenon of the rapid respiratory rate in individual mice was used as an auxiliary indicator. The mice were continuously administered for 7 days, and the time of intoxication and sobering up were observed and recorded [21].

### 2.5. Testing of Blood Indicators

The mice underwent consecutive gavaging for seven days based on their respective groupings. Thirty minutes following the final gavaging session, blood samples were obtained via ocular blood sampling. These samples were then centrifuged at 3000 revolutions per minute for 10 min in sodium heparin-coated centrifuge tubes. The supernatant from the centrifuged blood was subsequently analyzed to determine ethanol concentration (utilizing a microplate assay provided by Jiangsu Addison Biotechnology Co., Ltd., Yancheng, China). Additionally, alkaline phosphatase (ALP), aspartate aminotransferase (AST), and alanine aminotransferase (ALT) levels were assayed according to the manufacturer’s instructions. Physiological and biochemical indices were further quantified using a hematology analyzer [22].

### 2.6. Preparation of Paraffin Sections and H.E. Staining of Various Tissues of Mice

The tissue samples were fixed in 4% paraformaldehyde fixative for 24 h. The tissues were gently rinsed with running water to ensure complete fixative removal and then dehydrated using ethanol in a gradient. After dehydration, the tissues were embedded in paraffin wax, and the embedded tissues were sliced and patched on a microtome and then baked at 37 °C in a constant-temperature oven. The baked sections were stained with hematoxylin and eosin. Finally, neutral gum was used to seal the areas, and the paraffin sections after H.E. staining were observed using an orthogonal microscope [23,24].

### 2.7. Detection of Antioxidant Indicators (SOD, GSH) and Inflammatory Factors (IL-1β, TNF-I, IL-6α)

After detecting total protein, SOD activity and GSH content were measured. Tissue was homogenized and centrifuged, and the supernatant was collected. Part of the supernatant was diluted to 1% and incubated with reagents at 37 °C for 30 min. Absorbance was read at 562 nm to calculate protein concentration. The remaining supernatant was incubated with reagents at 37 °C for 20 min, and SOD activity was calculated from absorbance at 460 nm. GSH content was determined from absorbance at 405 nm after incubating tissue homogenate with reagents at 37 °C for 5 min. Tissue samples were homogenized with PBS and centrifuged, and the supernatant was collected. After incubating with biotinylated antibody and enzyme conjugate at 37 °C, *IL-1β*, *TNF-I*, and *L-6α* levels were calculated from the standard curve based on absorbance at 450 nm [25,26,27].

### 2.8. Measurement of Sperm Viability and Related Motility Indexes in Mice

The spermatozoa in the epididymis of each group of mice were collected and diluted with diluent, 2 μL of spermatozoa was dropped onto eight-chamber slides using a pipette gun, and the spermatozoa viability and related motility indexes were measured using a sperm analyzer (Minitube International, Inc., Tiefenbach, Germany) [28].

### 2.9. RNA Transcriptome Sequencing (RNA-Seq)

Based on the Illumina High-Throughput Sequencing Platform (Agilent Technologies, Inc., Waldbronn, Germany), transcriptome sequencing was conducted on three mice from each group, with a total of five samples analyzed. The sequencing process encompassed RNA extraction, RNA detection, library construction, and on-board sequencing. As a result, a total of 143.24 Gb of clean data was acquired, with each sample yielding 7 Gb of clean data and a Q30 base percentage of 92% or higher [29,30].

### 2.10. Differential Gene Screening

DESeq2 was utilized for differential expression analysis between sample sets with biological duplicates, aiming to identify the set of differentially expressed genes across two distinct biological conditions. Additionally, feature counts was employed to tally the reads of the genes. Following the differential analysis, the False Discovery Rate (FDR) was determined by adjusting the multiple hypothesis testing probabilities (*p*-value) through the Benjamini–Hochberg method. Differential genes were then filtered based on a log2 fold change ≥ 1 and an FDR < 0.05 [31,32,33].

### 2.11. RNA Extraction and Reverse Transcription

mRNA was extracted using the TRlzol method [34]. The mRNA extracted from tissue samples was then strictly reverse transcribed using the PrimeScript™ RT Master Mix (Perfect Real Time) kit (TaKaRa Bio Inc., Dalian, China) (Table S2).

### 2.12. Real-Time Quantitative PCR

Using Primer (5.0) software, primers were designed based on reference sequences from NCBI (https://www.ncbi.nlm.nih.gov, accessed on 10 January 2024) and subsequently synthesized by Sangon Biotech Co., Ltd. (Shanghai, China). Glyceraldehyde-3-phosphate dehydrogenase (*GAPDH*) was selected as the internal reference gene for real-time quantitative PCR, with three technical replicates. Real-time quantitative PCR was achieved using a fluorescent quantitative PCR detection system (BIO-RAD, Hercules, CA, USA) [35,36,37]. The relative gene expression levels were calculated using the 2^−∆∆Ct^ method (Tables S3 and S4).

### 2.13. Statistical Analysis of Data

The one-way ANOVA analysis was performed using SPSS 22.0 software. Significance testing was conducted using the t-test method, and significant results were denoted using the asterisk annotation system. The results are expressed as ‘mean ± standard error’, where a single asterisk (*) indicates a significant difference at *p* < 0.05, while a double asterisk (**) signifies a highly significant difference at *p* < 0.01 [38].

## 3. Results

### 3.1. Determination of the Optimal Level of Intoxication for Liquor

A gavage dose of 0.16 mL/10 g body weight was determined to cause 100% intoxication without any fatalities in two separate gradient tests. Consequently, a dose of 0.16 mL/10 g was selected for the subsequent testing, as detailed in Table 1 and Table 2.

### 3.2. Determination of the Optimal Koumiss Dose

Low-dose (LL), medium-dose (ML) and high-dose (HL) koumiss gavage groups were set up, and after 7 days of continuous gavage, the ML group showed the lowest mortality rate, which was close to that of the blank control group. Therefore, 0.10 mL/10 g was determined as the optimal koumiss gavage dose (Table 3).

### 3.3. Effect of Koumiss on Behavioral Indices in Intoxicated Mice

Significant differences were observed in the intoxication time, sobriety time, and mortality rate of mice across various groups. Specifically, the intoxication time of mice in the KL group was notably longer compared to the other groups, while their sobriety time was the shortest. Furthermore, apart from group B, the KL group exhibited the lowest mortality rate (Table 4). These findings suggest that the administration of koumiss has the potential to mitigate the intoxicating effects and harmful impact of alcohol to a certain degree, thereby exerting an anti-intoxication and protective effect.

### 3.4. Effect of Koumiss on the Blood of Intoxicated Mice

**Figure 1:**
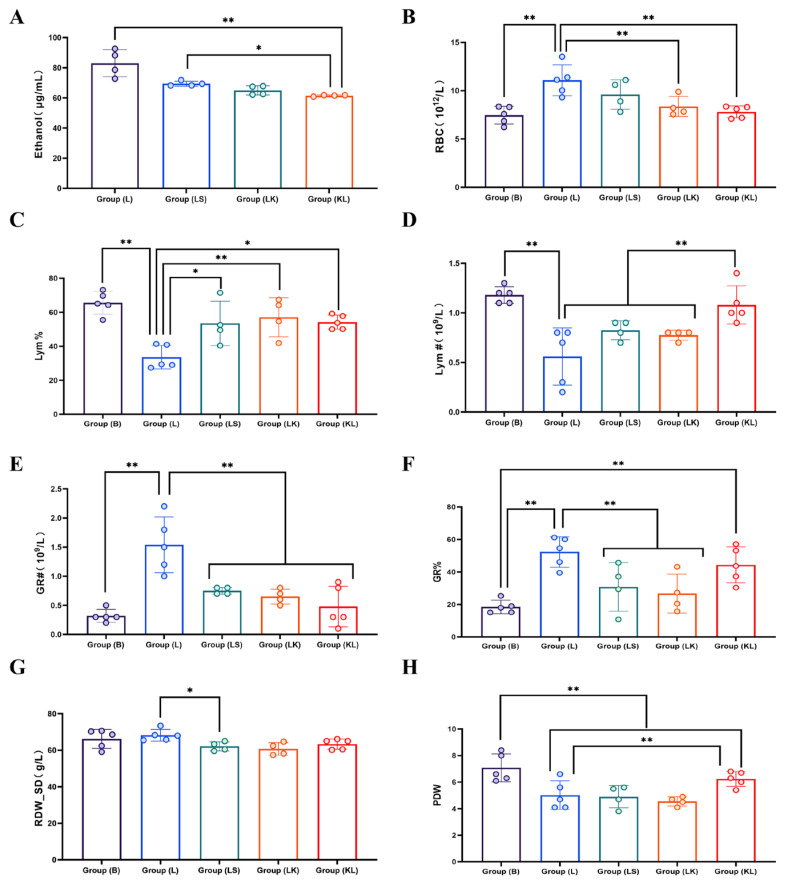
Alcohol content and blood physiological indexes, * indicates *p* < 0.05; ** *p* < 0.01. (**A**) Blood ethanol content comparison among groups; (**B**) RBC count across different groups; (**C**) Percentage of lymphocytes (Lym%) in groups; (**D**) Lymphocyte count (Lym#) among groups; (**E**) Granulocyte count (GR#) across groups; (**F**) Percentage of granulocytes (GR%) in groups; (**G**) RDW-SD variations across different groups; (**H**) PDW variations among groups.

After the administration of liquor, the ethanol content in the blood of mice escalated significantly. Compared to group L, the ethanol content in groups LS, LK, and KL decreased significantly (*p* < 0.01). Among them, the ethanol content in group KL was notably lower than that in group LS (*p* < 0.05) (Figure 1A). Furthermore, distinct variations were observed in the blood physiological indexes, including RBC, Lym#, GR#, Lym%, GR%, RDW-SD, and PDW, among the different groups (Figure 1B–H). These findings suggest that koumiss possesses the capability to attenuate the rate of increase in blood alcohol content and mitigate the deleterious effects of intoxication on the blood physiology of mice.

### 3.5. Effect of Koumiss on the Liver of Intoxicated Mice

**Figure 2:**
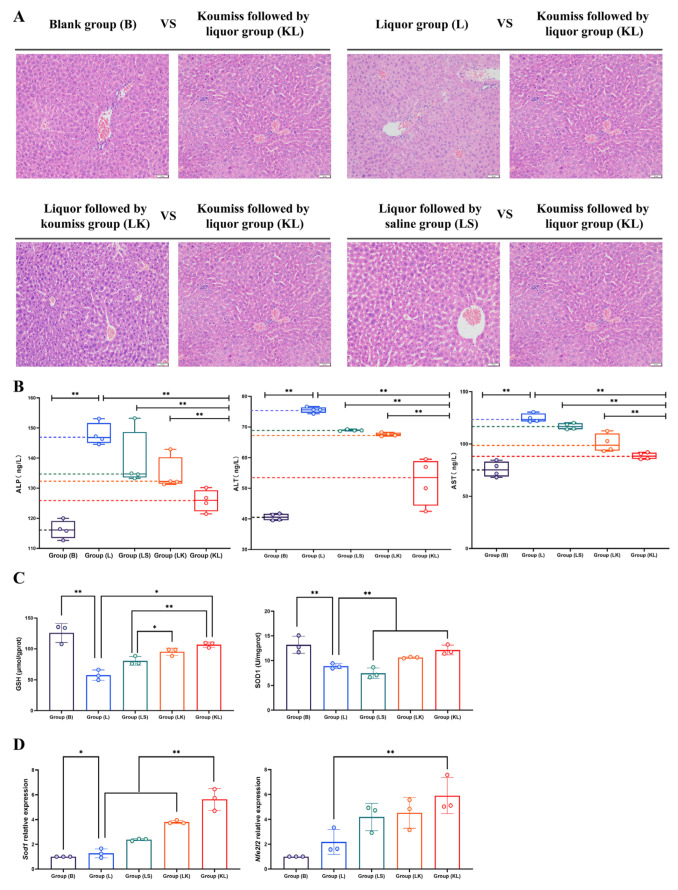
Liver sections and associated indexes, * indicates *p* < 0.05; ** *p* < 0.01. (**A**) Liver sections stained with H.E., magnification 20×; (**B**) Concentration levels of ALP, ALT, and AST; (**C**) Levels of SOD and GSH in the liver tissue; (**D**) Relative expression of *Sod1* and *Nfe2l2*.

Tissue sections revealed distinct differences among the groups. In group B, the liver lobule structure appeared clear, with a uniform and dense population of hepatocytes, most of which exhibited normal morphology. However, in group L, inflammatory cell infiltration was observed in the confluent area, accompanied by swollen hepatocytes, blurred intercellular borders, punctate necrosis in some hepatocytes, a disrupted liver lobule structure, visible rounded vacuoles in the intercellular cytoplasm, and evidence of hepatocyte fat metaplasia. Group LK exhibited similar but less-severe changes, including minor inflammatory cell infiltration, hepatocyte swelling, blurred intercellular borders, and point-like necrosis in some hepatocytes. In contrast, group KL showed only slight hepatocyte edema with a somewhat loose cytoplasm. The LS group presented with minor inflammatory cell infiltration in the confluent area, swollen hepatocytes, blurred intercellular borders, point-like necrosis in some hepatocytes, and a blurred hepatic lobule structure (Figure 2A).

Clinically, ALP, ALT, and AST are key indicators for assessing liver injury [39,40,41]. Notably, group L exhibited the highest levels of these three markers, with highly significant differences compared to groups B and KL. The levels in groups B and KL were relatively similar (Figure 2B). Regarding antioxidant factors in liver tissue, SOD and GSH levels were significantly higher in group B compared to group L, with group KL levels closely resembling those of group B (Figure 2C). Furthermore, *Sod1* and *Nfe2l2*, critical transcription factors regulating antioxidant stress, showed highly significant expression levels in group KL (Figure 2D). Specifically, the expression level in the KL group was remarkably higher than that in the L group, while the B group was comparable.

### 3.6. Effect of Koumiss on the Gastric Tissue of Intoxicated Mice

**Figure 3:**
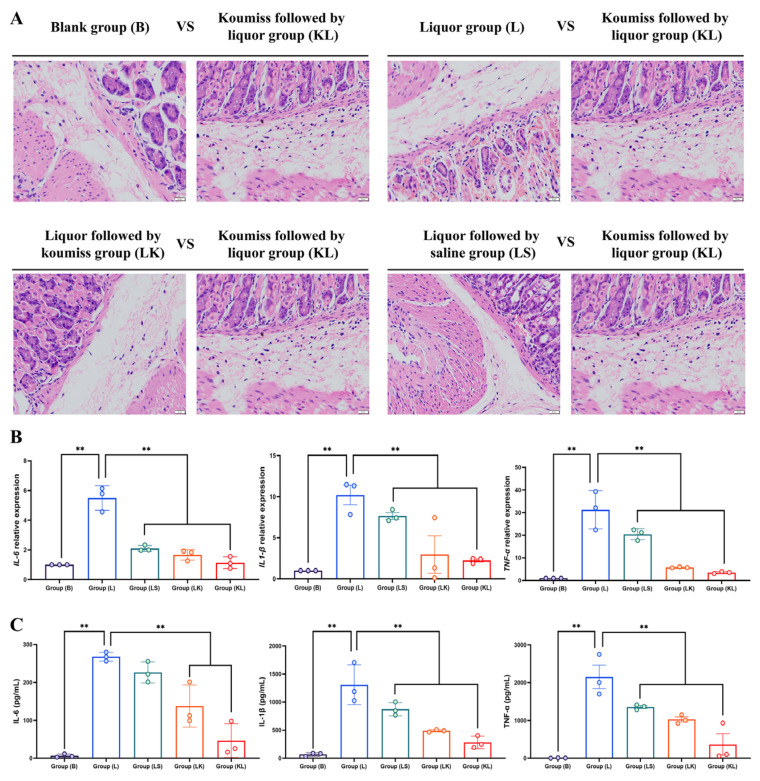
Gastric sections and associated indexes, ** *p* < 0.01. (**A**) Gastric sections stained with H.E., magnification 40×, arranged from left to right: group B, group L, group LS, group LK, and group KL; (**B**) Relative expression of *Il-6*, *Il-1β*, and *Tnf-α*; (**C**) Levels of Il-6, Il-1β, and Tnf-α in gastric tissue.

Gastric tissue sections examined under the microscope revealed distinct differences among the groups. In group B, a clear boundary between the muscular and mucous layers was observed, with tightly arranged primary and wall cells, and a smooth, flat mucous epithelium. However, in group L, severe damage was evident in the upper mucous layer and lamina propria, accompanied by a significant infiltration of red blood cells into the mucous layer and numerous crater-like lesions. The damage was more pronounced in group LK, where the upper mucous layer suffered greater destruction, with some red blood cell infiltration and a few crater-like lesions present. By contrast, the KL group exhibited a relatively intact upper mucosal layer, with only minimal hemoglobin cell infiltration into the mucosal tissue layer. In the LS group, severe damage to the upper mucosal layer was noted, while the lamina propria remained relatively unscathed. A large number of hemoglobin cells had infiltrated the mucosal tissue layer, resulting in numerous crater-like injuries and the partial lysis of muscle fibers (Figure 3A).

*TNF-α*, *IL-1β*, and *IL-6* are key pro-inflammatory cytokines [42,43,44]. Both fluorescence quantification and ELISA results indicated similar expression trends for *TNF-α*, *IL-1β*, and *IL-6* across all groups. Notably, group L demonstrated significantly higher expression levels compared to the other groups, whereas the expression level in group KL was the closest to that observed in group B (Figure 3B,C).

### 3.7. Effect of Koumiss on the Gastric Tissue of Intoxicated Mice

**Figure 4:**
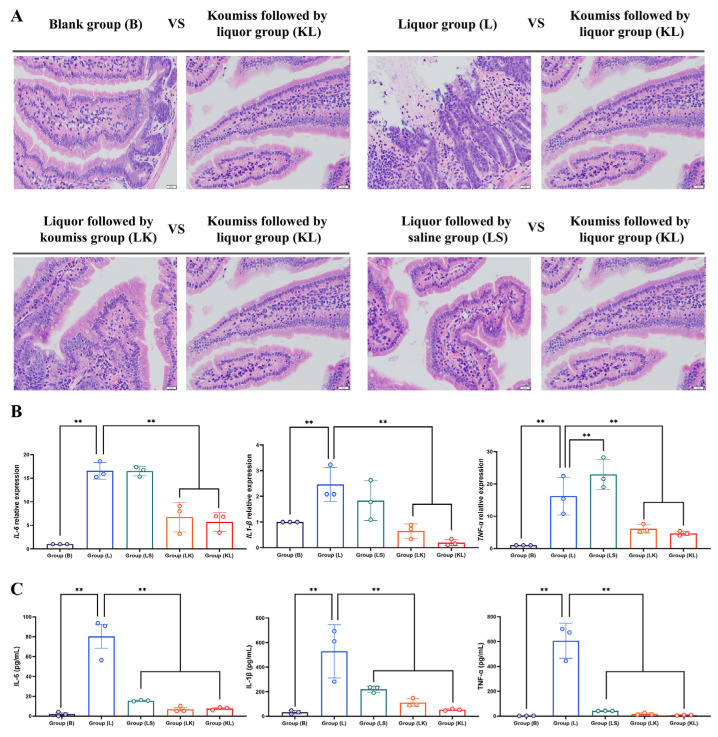
Intestinal sections and associated indexes, ** *p* < 0.01. (**A**) Intestinal sections stained with H.E., magnification 40×, arranged from left to right: group B, group L, group LS, group LK, and group KL; (**B**) Relative expression of *Il-6*, *Il-1β*, and *Tnf-α*; (**C**) Levels of Il-6, Il-1β, and Tnf-α in intestinal tissue.

In the small intestine tissue of group B, the single-layer columnar epithelial cells were tightly arranged alongside the cells of the mucosal muscle layer and the lamina propria, exhibiting a clearly defined structure. No inflammatory cell infiltration was observed between the tissues. Conversely, group L exhibited damage to the intestinal mucosa, with disrupted columnar epithelial cells and a significant infiltration of red blood cells between tissue layers. Group LK sections revealed mild intestinal mucosa damage, accompanied by a minor infiltration of red blood cells between layers. In contrast, group KL demonstrated a relatively intact intestinal mucosa with minimal red blood cell infiltration within the mucosal tissue layers. Similarly, the intestinal mucosa of the KL group remained largely unaltered, with only a scant presence of hemoglobin cells in the mucosal tissue layers. The LS group showed slight damage to the upper layer of the intestinal mucosa, while the lamina propria remained relatively intact, albeit with some hemoglobin cell infiltration between tissue layers (Figure 4A).

The results of the pro-inflammatory cytokine assay indicated similar expression trends for *IL-1β* and *IL-6* at the RNA level. Notably, the L group exhibited significantly higher expression compared to the B and KL groups. On the other hand, *Tnf-α* expression was highest in the LS group, although it remained considerably elevated in the L group compared to the B and KL groups (Figure 4B). The ELISA assay for IL-6, IL-1β, and Tnf-α revealed a comparable expression trend, with the L group demonstrating significantly higher levels than the other two groups. Specifically, the L group’s presentation was notably elevated compared to the others, while the KL group closely resembled the B group (Figure 4C).

### 3.8. Effects of Koumiss on Testicular Morphology in Intoxicated Mice

**Figure 5:**
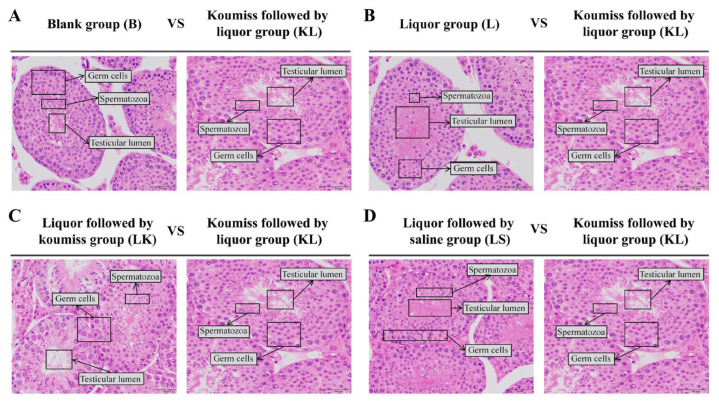
Morphological changes of mouse testis (H.E.). (**A**) Comparison of paraffin sections and H.E. staining of testicular tissue of drunken mice in group B and group KL. (**B**) Comparison of paraffin sections and H.E. staining of testicular tissue of drunken mice in group L and group KL. (**C**) Comparison of paraffin sections and H.E. staining of testicular tissue of drunken mice in group LK and group KL. (**D**) Comparison of paraffin sections and H.E. staining of testicular tissue of drunken mice in group LS and group KL.

The results of paraffin sections and H.E. staining of the testes of intoxicated mice showed that (Figure 5) the testicular tissue morphology and structure, spermatogenic cell density, and number of spermatozoa in the lumen of the tubules were similar in group B compared with group KL, and no obvious abnormality was observed (Figure 5A). The spermatogenic cell density of groups L, LK, and LS were smaller, and the spermatozoa in the lumen of the tubules were fewer in the respective groups compared with group KL (Figure 5B–D).

### 3.9. Effects of Koumiss on Sperm Motility and Related Motility Indexes in Intoxicated Mice

As shown in the statistical results of sperm motility and related motility indexes in different groups of drunken mice (Table 5), the sperm motility, linear motion, fast motion and ring motion of groups B and KL were significantly higher than those of group L (*p* < 0.01), and rest was significantly lower than in group L (*p* < 0.01). Group B had no highly significant difference in sperm motility, linear motion, slow motion, in-place motion and rest compared to group KL (*p* > 0.01).

### 3.10. Effect of Koumiss on Testicular Differential Genes in Intoxicated Mice

**Figure 6:**
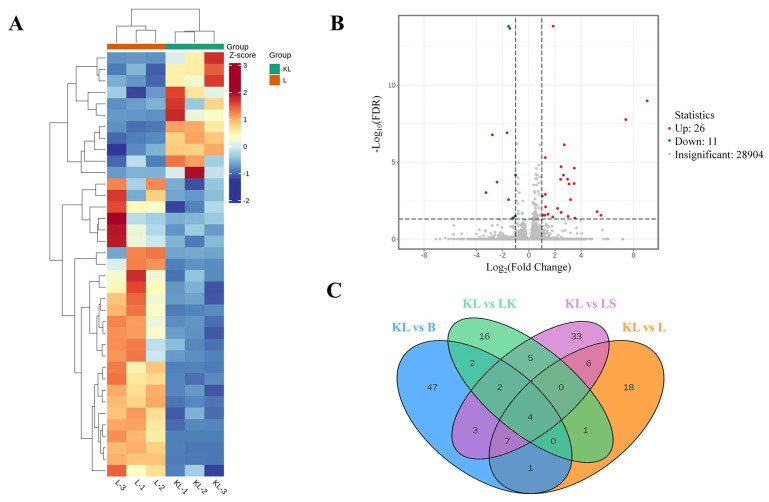
Related results of differential analysis from transcriptome sequencing of drunken mice testis. (**A**) Differential gene clustering heat map; the abscissa represents the sample name and hierarchical clustering results, and the ordinate represents the differential gene and hierarchical clustering results; Red indicates high expression and blue indicates low expression. (**B**) Differential gene volcano map; the abscissa represents the change of gene expression multiples, and the ordinate represents the significance level of differential genes. (**C**) Different groups of differential gene Wayne diagram. The non-overlapping region of the Wayne diagram represents the unique differential genes of the differential group, and the overlapping region represents several overlapping differential groups.

The correlation results of the post-sequencing differential analysis of the testicular transcriptome of intoxicated mice showed that the genes in the L group and the KL group had a high degree of differentiation (Figure 6A). Among them, a total of 66 differential genes (up: 19, down: 47) were screened in the KL group compared with the B group; 37 differential genes (up: 11, down: 26) were screened in the KL group compared with the L group (Figure 6B); 30 differential genes (up: 14, down: 16) were screened in the KL group compared with the LK group; and there was a total of 60 differential genes (up: 20, down: 40), while 4 differential genes that were screened existed across different groups, namely *mt-Atp6*, *mt-Co3*, *Gm8229*, *Gm3543* (Figure 6C).

### 3.11. Differential Gene GO, KEGG Analysis

**Figure 7:**
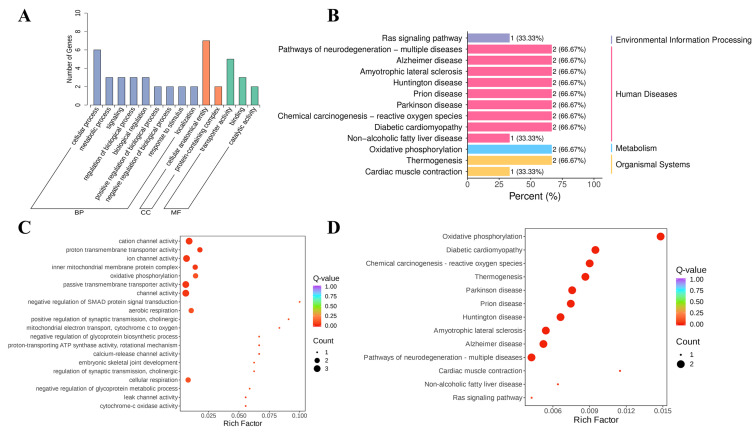
Differential gene GO and KEGG analysis. (**A**) Differential gene secondary entry classification diagram; the abscissa represents the secondary GO entry, the ordinate represents the number of differential genes on the GO entry annotation; (**B**) The KEGG enrichment histogram; the abscissa represents the number of differentially expressed genes annotated to the pathway, and the ordinate represents the name of the KEGG pathway. Number in the figure; (**C**) GO enrichment scatter diagram of differentially expressed genes; ordinate represents GO entries, abscissa represents Rich factor. The greater the Rich factor, the greater the degree of enrichment. The larger the point, the more the number of differential genes enriched in the pathway. The redder the color of the point, the more significant the enrichment; (**D**) KEGG enrichment scatter plot; ordinate represents KEGG pathway, abscissa represents Rich factor. The greater the Rich factor, the greater the degree of enrichment. The larger the point, the more the number of differential genes enriched in the pathway. The redder the color of the point, the more significant the enrichment.

The second-level classification statistical results of GO annotation of differential genes can be seen in Figure 7A. The second-level GO entries with a significant enrichment of differential genes in the KL group and L group include ‘cellular anatomical entity’, ‘protein-containing complex’, ‘transporter activity‘ and other entries in the biological process (BP); ‘cellular anatomical entity ‘and ‘protein-containing complex‘ in the cellular component (CC); and the ‘transporter activity’, ‘binding’, and ‘catalytic activity‘ entries in the molecular function (MF). The results of GO enrichment analysis of differential genes can be seen in Figure 7C. The differentially expressed genes in the KL group and L group were significantly enriched in ‘cation channel activity’, ‘passive transmembrane transporter activity’, ‘oxidative phosphorylation’, ‘inner mitochondrial membrane protein complex’, ‘ion channel activity’, ‘proton transmembrane transporter activity‘ and other items. The results of KEGG enrichment analysis showed that in the Environmental Information Processing, Human Diseases, Metabolism and Organismal Systems sections, the differential genes of the KL group and L group were enriched to 1,9,1 and 2 pathways, respectively (Figure 7B). The results of KEGG enrichment analysis are graphically displayed, which were mainly enriched in ‘Oxidative phosphorylation’, ‘Diabetic cardiomyopathy’, ‘Chemical carcinogenesis-reactive oxygen species’, ‘Thermogenesis, Parkinson disease’, ‘Prion disease’, ‘Huntington disease‘ and other pathways (Figure 7D).

### 3.12. Differential Gene GSEA Analysis

**Figure 8:**
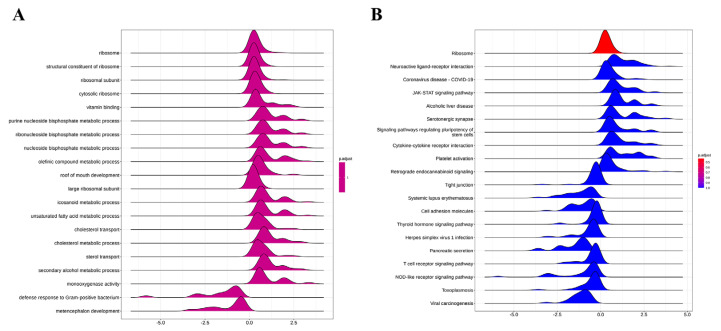
Differential gene GSEA analysis. (**A**) The GO database GSEA enrichment ridge map; the abscissa is the enrichment score (ES), and the ordinate is the enriched pathway. The ES value greater than 0 indicates that the pathway is activated, and the enriched core genes are upregulated genes. The ES value less than 0 indicates that the pathway is inhibited, and the enriched core genes are downregulated genes; (**B**) The KEGG database GSEA enrichment ridge map; the abscissa is the enrichment score (ES), and the ordinate is the enriched pathway. The ES value greater than 0 indicates that the pathway is activated, and the enriched core genes are upregulated genes. The ES value less than 0 indicates that the pathway is inhibited, and the enriched core genes are downregulated genes.

The results of the GSEA analysis of differential genes can be seen in Figure 8 for the GSEA analysis of GO data sets. Items such as ‘ribosome’, ‘structural constituent of ribosome’, ‘ribosomal subunit’, ‘cytosolic ribosome’, ‘vitamin binding’, ‘purine nucleoside bisphosphate metabolic process’, ‘ribonucleoside bisphosphate metabolic process’, ‘nucleoside bisphosphate metabolic process ‘and ‘olefinic compound metabolic process‘ were significantly enriched as upregulated, while ‘defense response to Gram-positive bacterium ‘and ‘metencephalon development‘ were significantly downregulated (Figure 8A) in the GSEA analysis of KEGG data sets. Pathways such as ‘Ribosome’, ‘Neuroactive ligand-receptor interaction’, ‘Coronavirus disease-COVID-19’, ‘JAK-STAT signaling pathway’, ‘Alcoholic liver disease’, ‘Serotonergic synapse’, and ‘Signaling pathways regulating pluripotency of stem cells‘ were significantly enriched as upregulated, while ‘Tight junction’, ‘Systemic lupus erythematosus’, ‘Cell adhesion molecules’, ‘Thyroid hormone signaling pathway’, ‘Herpes simplex virus 1 infection’, ‘Pancreatic secretion’, and ‘T cell receptor signaling pathway‘ were significantly enriched as downregulated (Figure 8B).

### 3.13. Validation of Real-Time Fluorescent Quantitative PCR

**Figure 9:**
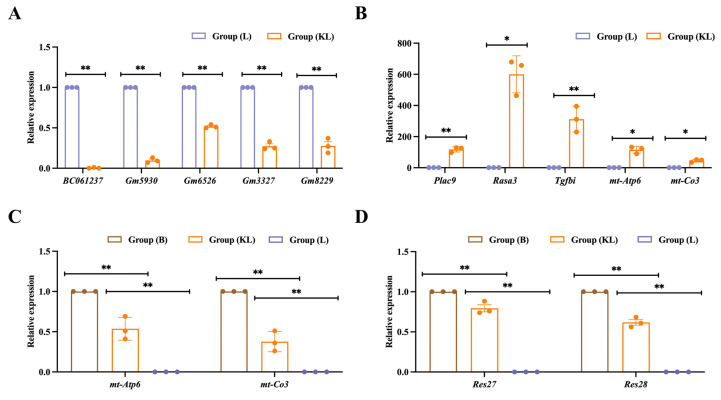
Quantitative real-time fluorescent PCR verification of key genes in testicular tissue and spermatozoa of alcohol-intoxicated mic, * indicates *p* < 0.05; ** *p* < 0.01. (**A**) qRT-PCR verification of downregulated genes in testicular tissue of alcohol-intoxicated mice (KL vs. L group); (**B**) qRT-PCR verification of upregulated genes in testicular tissue of alcohol-intoxicated mice (KL vs. L group); (**C**) qRT-PCR verification of mitochondrial-related genes in spermatozoa of alcohol-intoxicated mice (KL vs. L group); (**D**) qRT-PCR verification of ribosome-related genes in spermatozoa of alcohol-intoxicated mice (KL vs. L group).

Quantitative real-time fluorescent PCR verification was conducted on key genes in testicular tissue of alcohol-intoxicated mice. Among the 37 differentially expressed genes identified between the KL group and the L group, five downregulated genes (*Plac9*, *Rasa3*, *Tgfbi*, *mt-Atp6*, *mt-Co3*) and five upregulated genes (*BC061237*, *Gm5930*, *Gm6526*, *Gm3327*, *Gm8229*) were selected for qRT-PCR analysis. The results indicated that *Plac9*, *Rasa3*, *Tgfbi*, *mt-Atp6*, and *mt-Co3* were significantly overexpressed in the KL group (*p* < 0.01), while *BC061237*, *Gm5930*, *Gm6526*, *Gm3327*, and *Gm8229* were significantly overexpressed in the L group (*p* < 0.01) (Figure 9A,B). Furthermore, quantitative real-time fluorescent PCR verification was performed on key genes in spermatozoa of alcohol-intoxicated mice. Specifically, the mitochondrial-related key genes *mt-Atp6* and *mt-Co3* were selected for qRT-PCR analysis, and the results showed that both *mt-Atp6* and *mt-Co3* were significantly overexpressed in the KL group (*p* < 0.01). Additionally, the ribosome-related key genes *Res27* and *Res28* were also selected for qRT-PCR analysis, and the results indicated that both *Res27* and *Res28* were significantly overexpressed in the KL group (*p* < 0.01) (Figure 9C,D).

## 4. Discussion

### 4.1. Protective Effects of Koumiss against Alcohol-Induced Toxicity in Mice: Focus on Blood Indices, Organ Damage, and Inflammatory Responses

Alcohol is a pro-neurotoxicant that affects the central nervous system far more significantly than other organs [45]. In the case of excessive alcohol consumption, it can seriously affect a person’s memory and judgment [46]. After a long period of excessive alcohol consumption, it can also cause more serious mental disorders and other diseases [47]. The long-term intake of large amounts of alcohol can cause damage to the central nervous system, which can also lead to psychological problems. Alcohol also affects brain coordination, leading to a greater likelihood of falling and accidents when intoxicated, which in severe cases can lead to central nervous system dysfunction, respiratory function paralysis, and even death [48,49,50]. When the intoxicating dose of liquor was instilled, the mice gradually lost their mobility, and increasing the dose of liquor by gavage caused an increase in the mortality rate. Therefore, the optimal liquor gavage dose for this test needs to be established for this alcohol’s harmful effect. Based on the combined consideration of the intoxication and mortality rate, the gavage dose of 0.16 mL/10 g was taken as the optimal intoxicating liquor gavage dose.

The results of the present experiment showed that intoxication increased the number of erythrocytes in the blood of mice. Still, koumiss restored this to the average value. Lymphocytes, whose role is to recognize and attack pathogens that invade and protect the organism from diseases, are essential in the immune system [51]. This experiment showed that intoxication decreased the number and percentage of lymphocytes in mouse blood, while sour horse koumiss slowed the decrease in lymphocytes. Zhao [52] also found that sour horse koumiss could significantly increase the number of lymphocytes when he investigated the effect of koumiss on the immunomodulatory effect of rats, which was consistent with the results of the present study. This indicates that koumiss can increase the proportion of immune cells and show its activity. As for neutrophils, their excess can increase the chance of infection, leading to acute infections and inflammation, and circulating neutrophil levels in patients after chronic excessive alcohol consumption were significantly increased [53]. In this experiment, it was also found that liquor increased the number and percentage of neutrophils, while koumiss reduced them. As for platelets, low fractional width leads to anemia [54], and this experiment showed that intoxication decreases the fractional width of platelets in the blood. At the same time, koumiss slows down the decrease in the fractional width. In summary, blood indexes are affected by the excessive intake of liquor, and these effects are often adverse, while koumiss relieves such adverse effects. In contrast, the comparison order with the koumiss infusion did not show significant differences in the blood indices.

Acetaldehyde is the main metabolite of ethanol, which has a strong peroxidizing effect on lipids and results in particularly pronounced damage to the liver, resulting in decreased liver and myocardial function [55]. It has been found that excessive alcohol consumption significantly increases the risk of alcoholic liver disease [56]. Studies have shown that the prevalence of alcoholic liver disease among adolescents and young adults has steadily increased over the past few decades to 2% (1988–1994), 4% (1999–2004), and 5% (2007–2012), respectively [57]. In this experiment, after the gavage of koumiss, the cellular structure in these organs was protected, and the damage was partially alleviated compared to the L group. As for the description of liver function, ALP (alkaline phosphatase), ALT (alanine aminotransferase), and AST (aspartate aminotransferase) are three common liver function indicators [58]. ALP is found mainly in the liver, bile ducts, and bones, and when the liver or bile ducts are damaged, ALP levels are elevated [59]; ALT and AST are found mainly in liver cells, and when liver cells are damaged, they are released into the bloodstream, resulting in elevated levels [60]. Therefore, these indicators can be used to detect liver diseases such as hepatitis, cirrhosis, and hepatocellular carcinoma. In this trial, it was found that all three indicators in group L significantly increased compared with group B. In comparison, koumiss decreased these three indicators, which was consistent with the results of the sections. The effect of koumiss first was more favorable, and the reason for the difference caused by this gavage order needs to be explored further in subsequent trials. As for alcoholic liver injury, although the pathogenesis is very complex, and the influencing factors include genetics, nutrition, environment, time, and other factors, free radical-mediated oxidative stress injury (oxidative stress) is the current mainstream view of the alcoholic liver injury mechanism [61]. In the liver, ethanol is first converted to acetaldehyde by the catalytic action of ethanol dehydrogenase (alcohol dehydrogenase, ADH) and then to acetic acid by the action of acetaldehyde dehydrogenase (acetaldehyde dehydrogenase, ALDH) [62].

During ethanol metabolism, there is an increase in the production of reactive oxygen radicals (reactive oxygen species (ROS)), which are the main cause of oxidative stress [63]. A small amount of free radicals exists in the normal organism, and these free radicals can be scavenged by antioxidant substances such as superoxide dismutase (SOD) and reduced glutathione (GSH). However, when ROS are produced in large quantities, the antioxidant substances are depleted, leading to an imbalance between oxidation and antioxidation, which triggers lipid peroxidation. ROS react with unsaturated fatty acids in the biological membranes, leading to a decrease in the unsaturated fatty acids of the membranes, which increases the permeability of the membranes and the damage to the mitochondria of the hepatocytes, ultimately leading to damage to the hepatocyte structure and function. At the same time, the toxic effect of acetaldehyde can reduce the GSH level and enhance the free radicals’ toxicity, thus triggering lipid peroxidation and promoting apoptosis [64]. SOD can prevent lipid peroxidation damage of the biological membranes and protect proteins and DNA from the ROS damage [65]. The results of the present study also verified that liquor reduced SOD and GSH levels in mice. Koumiss alleviated this trend of antioxidant reduction, and likewise, for the relative expression of *Sod1* at the mRNA level, there was a change in this trend. Similarly, Nrf2 is an important intracellular antioxidant that regulates intracellular oxidative stress and protects cells from oxidative damage. The expression of Nrf2 is regulated by various factors, including oxidative stress, inflammation, drugs, etc. [66]. In the present assay, *Nfe2l2*, a gene that encodes the transcription factor Nrf2, showed a significantly lower relative expression in group L than in group B. In contrast, the gavage of sour horse milk will increase its relative expression. Compared with the sequence of gastric koumiss infusion, the above results were significantly different, and the related mechanism needs to be further explored in subsequent experiments.

When drinking alcohol for a long time and in large quantities, alcohol will vigorously stimulate the gastric mucosa, causing damage to the gastric mucosa cells and increasing the secretion of gastric acid. This makes it easy for gastritis, ulcers, and other diseases to manifest [67]. Long-term heavy drinking will also increase the risk of gastric cancer because alcohol can damage the stomach cells, making the cells abnormal, and even malignant tumors [68]. When the human body ingests 40% of alcohol in a single dose, it will lead to constipation, gastric mucosal edema, and desquamation, as well as generalized edema, fibrin deposition, microvascular rupture, and photomicrographic extensive lamina propria edema of the epithelium. At the same time, it is mainly the microvascular structure of the gastric mucosa that is acutely damaged by alcohol. The pathological sections and scores of gastric tissues in this experiment also fully confirmed the above damage. The damage to the upper and lamina propria of the gastric mucosa after the gavage of liquor was severe, with a large number of red blood cells infiltrating the mucosal tissue layer, and there was a large number of crater-like damages. In the group with koumiss gavage, the above injuries became less severe to a certain extent, and the effect of gavage with koumiss first was more favorable. Pathological section scores were also in line with the above trend. Gastric tissue scores for all indicators in the KL group were significantly higher than those in the LK, LS, and L groups. As for the small intestinal tissues, alcohol affects the activity of enzymes in the small intestine, which can lead to weakened peristalsis and inhibit the absorption and transport function of the small intestinal epithelial cells, thus reducing the uptake and utilization of nutrients and affecting the digestion and absorption function, which can lead to nutritional deficiencies and dyspepsia [69]. Some aerobic bacteria in the gut contain alcohol hydrogenase, an enzyme that metabolizes alcohol and produces acetaldehyde in the stomach, leading to intestinal disorders and related symptoms. Alcohol can also directly damage small intestinal epithelial cells, leading to oxidation and the disruption of cell membranes, resulting in cellular dysfunction and death. The damage and death of these cells may trigger an inflammatory response and a disorder of the small intestinal mucosal barrier. A high intake of alcohol can alter the composition and number of florae in the small intestine, resulting in an increase in harmful bacteria and a decrease in beneficial bacteria, leading to an imbalance in the flora, which can further damage the small intestinal mucosal barrier [70]. Chronic alcohol consumption also increases the risk of small bowel cancer, of which alcohol is one of the main causative factors [71]. Alcohol also inhibits the activity of immune cells under the small intestinal mucosa, which affects immune function and reduces the resistance of the small intestine, making it susceptible to infection with pathogenic bacteria and inflammation [72]. The results of this experiment were confirmed by the pathological sections and scores of small intestine tissues, which showed significant damage to the intestinal mucosa following the gavage of liquor. The damage included the detachment of the mucosa, destruction of columnar epithelial cells, and infiltration of hemoglobin cells in the layers of the tissues. In contrast, the group given koumiss showed damage, and the protective effect was more pronounced when koumiss was given before liquor. The scores of the pathological sections were consistent with this trend. Compared to the LK and LS groups, the KL group had significantly higher scores for all indexes of gastric tissues. The experiments indicate that alcohol causes significant harm to both gastric and small intestinal tissues, primarily due to the damage to the mucosal structure. In contrast, gastric koumiss, particularly when taken before liquor, can slow the damage and offer protective benefits.

TNF-α (Tumor Necrosis Factor-α), IL-1β (Interleukin-1β), and IL-6 (Interleukin-6) are three cytokines that play an essential role in the immune system [73]. TNF-α is a cytokine produced by a wide range of cells; it plays a critical role in processes such as inflammation, immune response, and apoptosis [74]. IL-1β is a cytokine produced by monocytes and macrophages; it plays a vital role in inflammation, immune response, and cell proliferation [75]. IL-6 is a cytokine produced by various cells; it plays an essential role in inflammation, immune response, and cell proliferation [76]. These three cytokines play an indispensable role in the inflammatory response, and their overproduction may lead to an excessive inflammatory response, which may cause various diseases [77]. In the present study, we found that after a gavage of liquor, the levels of TNF-α, IL-1β, and IL-6 were significantly increased in the stomach and small intestine tissues of mice, indicating that alcohol intake can cause an inflammatory response. In contrast, the gavage of koumiss followed by liquor significantly reduced the expression levels of these three inflammatory factors. Meanwhile, the expression of mRNA levels of *TNF-α*, *IL-1β*, and *IL-6* inflammatory factors showed that the relative expression of *TNF-α*, *IL-1β*, and *IL-6* in the L group was significantly higher than that in the B group. In contrast, the expression level in the koumiss group was markedly lower than that in the L group, but the difference between the L group and the group consuming white liquor followed by koumiss was not significant. It is hypothesized that alcohol damages the gastric and small intestinal tissues mainly by increasing the expression level of inflammatory factors, which triggers the inflammatory process. In contrast, koumiss reduces relevant inflammatory factors and interferes with the inflammatory process, thus protecting the gastric and small intestinal tissues. However, why the sequence of a gavage of koumiss and liquor significantly affects inflammatory factor expression levels still needs to be further investigated in subsequent experiments. The damage caused by liquor to mice was mainly in the blood system and major organs (liver, stomach, and small intestine). Koumiss mitigated these damaging effects to a certain extent, but there was still a gap compared to the normal state. The gavage sequence of koumiss and liquor differed in the impact of some indicators, and the rationale for this needs to be investigated in further experiments.

### 4.2. Koumiss Mitigates Alcohol-Induced Toxicity in Mouse Testes by Modulating Mitochondrial and Ribosomal Functions

In the reproductive process of animals, the testes, as vital male reproductive organs, play a crucial role in androgen secretion and spermatogenesis [78,79]. Morphologically, the testes are paired oval structures encased by a tunica albuginea and tunica vaginalis. While most animals’ testes descend into the scrotum, a minority exhibit testicular ectopia within the abdominal cavity [80]. Normal testicular development is essential for spermatogenesis and sperm quality, with excessive ethanol consumption adversely affecting the testes and their cellular constituents. Studies by Ayodele et al. [81] and Siervo et al. [82] have shown that ethanol administration leads to significant loss of testicular germ cells in rats and morphological and physiological alterations in mammalian reproductive systems, including testicular remodeling, reduced sperm counts, and compromised sperm motility parameters. These findings underscore the close correlation between testicular architecture and sperm quality under ethanol exposure, significantly influencing its assessment. Our experiments, through testicular histological analysis and sperm motility assessments, validate these observations. Compared to the control group (B), the ethanol-treated group (L) exhibited abnormal seminiferous tubule structures, resulting in reduced sperm counts. Moreover, the B group demonstrated significantly higher sperm motility, linear motion, fast motion, and ring motion than the L group (*p* < 0.01). Notably, the koumiss-pretreated group (KL) displayed similar differences with the L group but no significant deviations from the B group, suggesting that koumiss can mitigate alcohol’s detrimental effects on mouse seminiferous tubules and sperm to some extent.

Spermatogenesis, the continuous production of sperm in male animals, is a dynamic and complex process occurring within the seminiferous tubules of the testes, involving various germ cells and somatic cells [83,84,85]. Mitochondria, the powerhouses of cells, generate ATP through oxidative phosphorylation, fueling various physiological activities in sperm cells [86,87,88]. During spermatogenesis, mitochondria not only supply energy but also participate in cytoskeleton formation and intracellular signaling, essential for spermatid differentiation and sperm motility [89,90,91,92,93]. Our transcriptomic analysis of testicular tissues from intoxicated mice identified differentially expressed genes related to mitochondrial respiration, *mt-Atp6* and *mt-Co3*, which were significantly downregulated in the L group, indicating alcohol’s impact on mitochondria through these genes. GO and KEGG enrichment analyses further emphasized the involvement of oxidative phosphorylation, inner mitochondrial membrane protein complexes, ion channel activity, and proton transmembrane transporter activity. qRT-PCR results confirmed the upregulation of *mt-Atp6* and *mt-Co3* in the KL group’s testicular tissues and semen, suggesting that koumiss alleviates alcohol’s reproductive toxicity by preserving mitochondrial respiratory function.

Ribosomes, critical for translating genetic codes into proteins, are essential components of sperm and play pivotal roles in spermatogenesis [94,95,96]. Research by Li et al. [97] highlights the involvement of specialized proteins in the ribosomal polypeptide exit tunnel, influencing sperm protein folding, stability, and function. Our GSEA analysis revealed the upregulation of ribosome-related terms, and qRT-PCR confirmed a significant expression of ribosomal genes *Res27* and *Res28* in the KL group’s semen compared to the L group. These findings suggest that alcohol also disrupts ribosomal functions in intoxicated mice, contributing to reduced fertility.

## 5. Conclusions

In conclusion, this comprehensive analysis of koumiss’s protective effects against alcohol-induced harm provides compelling evidence of its potential as a natural agent for mitigating the detrimental impacts of excessive alcohol consumption. Through a meticulous mouse model study, the research demonstrates that koumiss pretreatment delays inebriation, accelerates sobering, and reduces mortality, while also mitigating alcohol’s negative effects on the blood indices, liver, gastrointestinal tract, and reproductive system. The study further uncovers that koumiss exerts its benefits by influencing mitochondrial and ribosomal functions, particularly in the reproductive system, offering insights into its underlying protective mechanisms. These findings not only underscore the nutritional and medicinal values of koumiss but also open new avenues for future research and potential applications in addressing alcohol-related health issues.

## Figures and Tables

**Table 1:** The drunkenness rate and mortality rate of mice by intragastric administration of liquor—gradient one.

Number	Number of Mice/*n*	The Amount of Gavage (mL/10 g)	Ebriety Rate/%	Mortality/%
1	10	0.10	60	0
2	10	0.15	80	0
3	10	0.20	100	40
4	10	0.25	100	100
5	10	0.30	100	100

**Table 2:** The drunkenness rate and mortality rate of mice by intragastric administration of liquor—gradient two.

Number	Number of Mice/*n*	The Amount of Gavage (mL/10 g)	Ebriety Rate/%	Mortality/%
1	10	0.16	100	0
2	10	0.17	100	20
3	10	0.18	100	30
4	10	0.19	100	30

**Table 3:** The mortality rate of mice after intragastric administration of liquor and different doses of koumiss was compared.

Group	Number of Mice/*n*	Dosage of Koumiss (mL/10 g)	Mortality/%
B	10	0.00	10
L	10	0.00	40
LL	10	0.05	30
ML	10	0.10	10
HL	10	0.15	30

Note: The mortality of 10% in the group B is attributed to one mouse that died on the first day of feeding. This may be due to individual physical conditions and failure to adapt to the environment.

**Table 4:** Effect of koumiss on behavioral indexes of drunken mice.

Group	Numbers/*n*	Drunk Time/h	Sober-Up Time/h	Mortality/%
B	10	—	—	10
L	10	0.31 ^c^ ± 0.03	6.83 ^a^ ± 0.25	60
LS	10	0.29 ^c^ ± 0.06	6.73 ^a^ ± 0.27	40
LK	10	0.40 ^b^ ± 0.04	6.01 ^b^ ± 0.20	30
KL	10	0.51 ^a^ ± 0.05	5.79 ^b^ ± 0.38	20

Note: Shoulder markers with different letters indicate highly significant differences (*p* < 0.05).

**Table 5:** Effects of koumiss on sperm motility and related motility indexes in intoxicated mice.

Index	Group				
	B (*n* = 3)	KL (*n* = 3)	L (*n* = 3)	LK (*n* = 3)	LS (*n* = 3)
Sperm motility/%	73.93 ± 1.86 Aa	58.75 ± 8.71 ABb	20.74 ± 7.93 Dd	41.52 ± 7.98 BCc	36.55 ± 6.59 CDc
Linear motion/%	64.78 ± 5.69 Aa	51.88 ± 9.14 ABa	16.43 ± 7.17 Cc	34.48 ± 8.24 BCb	31.65 ± 8.53 BCb
Fast motion/%	10.03 ± 1.59 Aa	5.56 ± 1.69 Bb	2.08 ± 1.16 Cc	3.74 ± 0.45 BCbc	3.49 ± 0.53 BCbc
Slow motion/%	12.70 ± 7.99 Aab	18.53 ± 5.50 Aa	4.45 ± 0.75 Ab	8.34 ± 4.13 Aab	12.04 ± 12.24 Aab
Ring motion/%	42.04 ± 4.69 Aa	27.80 ± 5.40 Bb	9.89 ± 6.43 Cd	22.39 ± 4.41 BCbc	16.11 ± 4.71 BCcd
In-place motion/%	9.15 ± 4.58 Aa	6.87 ± 0.89 Aa	4.31 ± 0.79 Aa	7.04 ± 1.43 Aa	8.24 ± 6.94 Aa
Rest/%	26.07 ± 1.86 Dd	41.25 ± 8.71 CDc	79.26 ± 7.93 Aa	58.48 ± 7.98 BCb	63.45 ± 6.58 ABb

Note: Shoulder markers with different capital letters in the table indicate very significant differences (*p* < 0.01), and different small letters indicate significant differences (*p* < 0.05).

## Data Availability

The original contributions presented in the study are included in the article/Supplementary Material, further inquiries can be directed to the corresponding author.

## Supplementary Materials

The following supporting information can be downloaded at: https://www.mdpi.com/article/10.3390/foods13152344/s1, Table S1: Nutritional composition table of fermented mare’s milk (koumiss); Table S2: Reverse transcription reaction system; Table S3: Primer information; Table S4: Real-time fluorescence quantitative PCR reaction system.
